# Bidirectional Relationship Between Tuberculosis and Hypothyroidism: An 18-Year Nationwide Population-Based Longitudinal Cohort Study

**DOI:** 10.3389/fmed.2022.900858

**Published:** 2022-07-12

**Authors:** Li-Ting Cheng, Chi-Hsiang Chung, Chung-Kan Peng, Chin-Chung Shu, Shu-Yu Wu, Sheng-Huei Wang, Gwo-Jang Wu, Chang-Huei Tsao, Chien-An Sun, Wu-Chien Chien, Shih-En Tang

**Affiliations:** ^1^Division of Pulmonary and Critical Care Medicine, Department of Internal Medicine, National Defense Medical Center, Tri-Service General Hospital, Taipei, Taiwan; ^2^Department of Medical Research, National Defense Medical Center, Tri-Service General Hospital, Taipei, Taiwan; ^3^Taiwanese Injury Prevention and Safety Promotion Association, Taipei, Taiwan; ^4^National Defense Medical Center, School of Public Health, Taipei, Taiwan; ^5^Department of Internal Medicine, National Taiwan University Hospital, Taipei, Taiwan; ^6^National Defense Medical Center, Graduate Institute of Aerospace and Undersea Medicine, Taipei, Taiwan; ^7^National Defense Medical Center, Graduate Institute of Medical Sciences, Taipei, Taiwan; ^8^Department of Obstetrics and Gynecology, National Defense Medical Center, Tri-Service General Hospital, Taipei, Taiwan; ^9^Department of Microbiology and Immunology, National Defense Medical Center, Taipei, Taiwan; ^10^Department of Public Health, College of Medicine, Fu-Jen Catholic University, New Taipei City, Taiwan; ^11^Big Data Research Center, College of Medicine, Fu-Jen Catholic University, New Taipei City, Taiwan; ^12^National Defense Medical Center, Graduate Institute of Life Sciences, Taipei, Taiwan

**Keywords:** hypothyroidism, international classification of diseases, levothyroxine, longitudinal study, mycobacterium tuberculosis, risk factors, tuberculosis

## Abstract

Some antituberculosis agents may cause hypothyroidism, and thyroid hormones play a vital role in *Mycobacterium tuberculosis* infection. However, the relationship between tuberculosis (TB) and hypothyroidism has not been clearly established. Therefore, this retrospective, longitudinal cohort study aimed to investigate the association between these two diseases using the 2000–2017 data from the Taiwan's National Health Insurance Research Database. The hypothyroidism and TB cohorts were matched with the control group in a 1:4 ratio. Adjusted hazard ratios (aHRs) were assessed using Cox proportional hazards regression analysis in each cohort. In total, 3,976 individuals with hypothyroidism and 35 120 individuals with TB were included in this study. The risk of developing TB in patients with hypothyroidism was 2.91 times higher than that in those without hypothyroidism (95% confidence interval [CI], 1.50–3.65). The subgroup of thyroxine replacement therapy (TRT) had a 2.40 times higher risk (95% CI, 1.26–3.01), whereas the subgroup of non-TRT had a 3.62 times higher risk of developing TB than those without hypothyroidism (95% CI, 2.19–4.84). On the other hand, the risk of developing hypothyroidism in patients with TB was 2.01 times higher than that in those without TB (95% CI, 1.41–2.38). Our findings provide evidence that TB and hypothyroidism are interrelated. Thus, clinicians and public health authorities should monitor the association between these two diseases to reduce the relevant disease burden.

## Introduction

Tuberculosis (TB) is a severe communicable disease that is among the top 10 causes of death worldwide. According to the World Health Organization, ~10 million new cases and 1.3 million deaths were reported in 2020. Of all TB cases in 2020, the proportion of adult men, adult women, and children is 56, 33, and 11%, respectively (1). Despite achieving a microbiological cure, many survivors face post-TB sequelae, thus increasing the overall disease burden (2).

Taiwan became an aged society in 2018. There were 16,472 and 7,823 new TB cases in 2005 and 2020, respectively, and the incidence rate decreased from 72.5 to 33.2 per 100,000 population during this period. More than half of the new TB cases occur in the elderly population (age ≥ 65 years) since 2005. Besides, there were 460 TB-related deaths in 2020 and the cumulative reduction between 2005 and 2020 was 53.5 %. Since 2006, the coverage rate of directly observed treatment, short course has reached 100%, and ~70% of patients with bacteriologically-positive TB were treated successfully in 2018 (3). Between 2006 and 2013, hypertension (HTN), diabetes mellitus (DM), chronic obstructive pulmonary disease (COPD), cardiovascular disease, and cancer were identified as common comorbidities in patients with TB in Taiwan. The risk of death in patients with TB and any comorbidity was 2.12 times higher than that in those without comorbidities (4). The Taiwan Centers of Disease Control highlights preventive treatment of high-risk individuals and early diagnosis of TB, and the elimination of TB is expected to be completed gradually by 2035, echoing the World Health Organization End TB Strategy (1).

Hypothyroidism is characterized by decreased thyroid hormone production in the thyroid gland. It can be classified as primary (due to thyroid hormone deficiency) or secondary/tertiary (due to abnormal hypothalamic-pituitary function). The diagnosis of hypothyroidism depends heavily on laboratory testing because of variable non-specific clinical manifestations (5–7). Women are more likely to have hypothyroidism than men (8). More than 95% cases of hypothyroidism are of the primary type (9, 10). Overt hypothyroidism and subclinical hypothyroidism are two degrees of primary hypothyroidism, and the prevalence of these disorders ranges from 0.1 to 3% and 1.6 to 15% (5, 8, 11–15), respectively. Annually, ~2–5% of cases of subclinical hypothyroidism may progress to overt hypothyroidism (6). Although chronic autoimmune thyroiditis is the main cause of primary hypothyroidism (5), other possible causes include iatrogenic diseases, deficiency or excessive consumption of iodine, drugs, and infiltrative diseases (5, 16–18). The common mechanism of drug-induced hypothyroidism includes inhibition of thyroid hormone synthesis, decreased absorption of T4, increased T4 clearance, increased type 3 deiodination, destructive thyroiditis, suppression of TSH, and immune dysregulation. Common drugs can cause hypothyroidism including lithium, amiodarone, omeprazole, lansoprazole, interferon alfa, interleukin-2, tyrosine kinase inhibitors (sunitinib, sorafenib, imatinib, etc.), and checkpoint inhibitors (ipilimumab, pembrolizumab, nivolumab, etc.). Patients taking these drugs should monitor possible hypothyroid symptoms and serum TSH should be measured at least every 6 to 12 months (17).

Thyroid hormones modulate various immune system functions, including chemotaxis, phagocytosis, production of reactive oxygen species, and the release of cytokine release (19). Hypothyroidism may have a detrimental effect on the immune system and subsequently make patients vulnerable to infection. Recent studies have shown that thyroid hormone signaling plays a vital role in optimal immune response during *Mycobacterium tuberculosis* (Mtb) infection (20), which could be related to infection or drug-related hypothyroidism. Thyroid tuberculosis, which has a frequency of 0.1–0.4%, may cause hypothyroidism owing to extensive glandular destruction due to caseous necrosis (21). Infectious agents have been reported to trigger autoimmune thyroid diseases by possible mechanisms including molecular mimicry theory and bystander activation theory (22). Rifampin increases T4 clearance, possibly because of enhanced hepatic T4 metabolism and biliary excretion of iodothyronine conjugates. Vaidya (16) and Montanelli (17) reported that rifampin causes primary hypothyroidism and there were few case reports of rifampin-induced hypothyroidism, and most of them had underlying Hashimoto's Thyroiditis, and some of them without underlying thyroid disease. Prior systemic review and meta-analysis showed that ethionamide and para-aminosalicylic acid were the most frequently reported drugs associated with the occurrence of hypothyroidism (23) and these drugs can cause hypothyroidism by inhibiting thyroid hormone synthesis through a mechanism of iodine organification inhibition (24, 25).

These studies may partially explain the causal relationship between TB and hypothyroidism; however, evidence from longitudinal analysis is lacking. Therefore, we conducted a bidirectional, nationwide, population-based cohort study to investigate the association between TB and hypothyroidism.

## Materials and Methods

### Data Source

In 1995, the Taiwanese government adopted the National Health Insurance (NHI) programme, which covers the healthcare data of more than 99% of Taiwanese residents (26). The data analyzed in this study were obtained from the National Health Insurance Research Database (NHIRD). The Longitudinal Health Insurance Database 2005 (LHID2005) (27), which contains the original claims data for 2,000,000 patients randomly sampled from the NHI enrollees registered in 2005 (28).

Previous studies have reported that the data of the LHID are representative of the entire Taiwanese population, and the accuracy of the disease diagnoses have been validated (29–31). Personal identification information from the claims data was encrypted and anonymized to protect the privacy and security of the patients (27). This study was approved by the Institutional Review Board of the Tri-Service General Hospital (approval number B-111-01).

### Study Design and Population

To analyze the bidirectional relationship between hypothyroidism and TB, this study had two main purposes. Diagnoses of both diseases were designated using the International Classification of Disease, 9th Revision, Clinical Modification (ICD-9-CM) codes for the 2000–2015 period and the ICD-10-CM codes for the 2016–2017 period.

**Figure 1:**
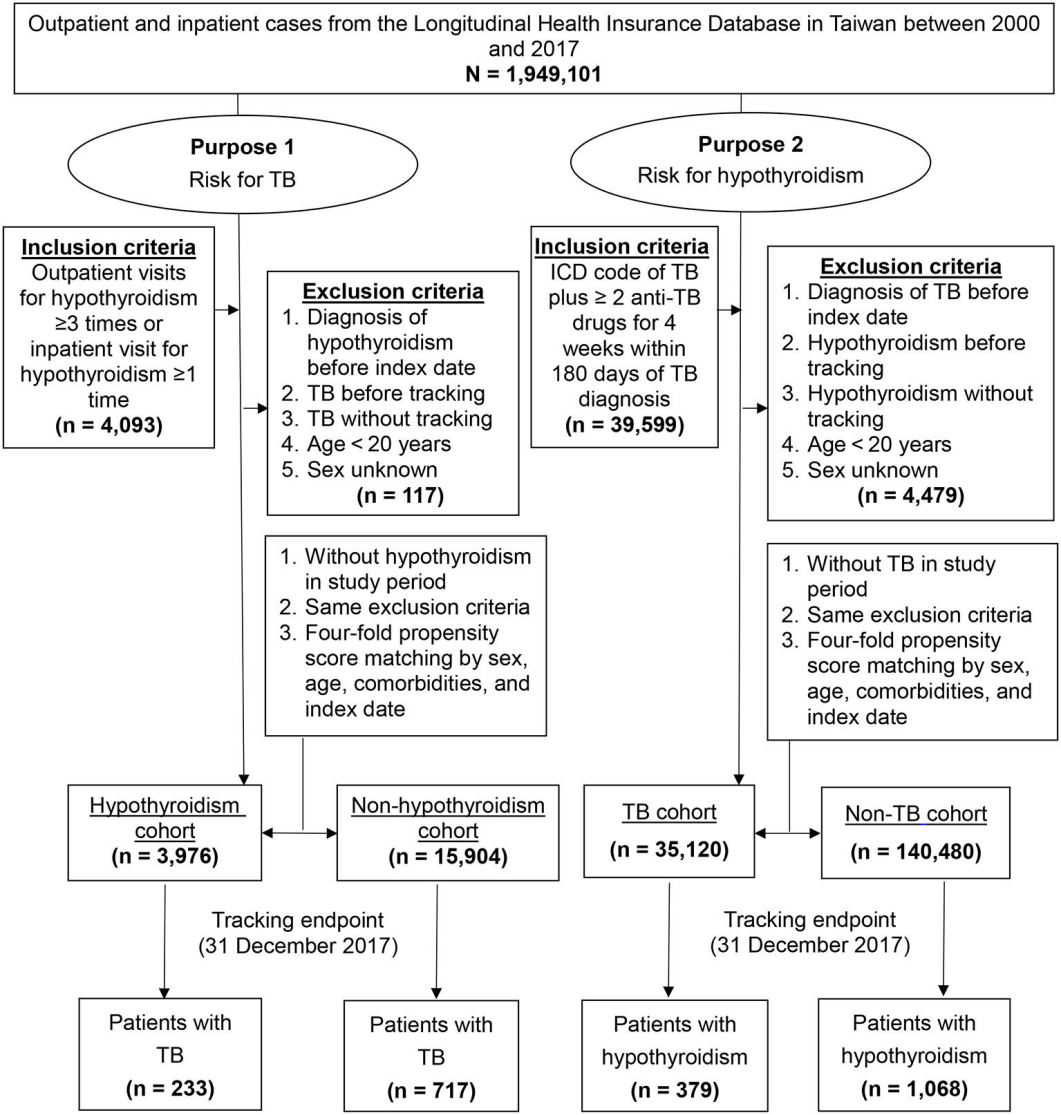
Flowchart of study participant selection. ICD, International Classification of Disease; TB, tuberculosis.

For purpose 1, we included patients with hypothyroidism (ICD-9: 243-244, ICD-10: E00, E01.8, E02-E03, E89.0) aged >20 years who had at least three outpatient visits or one hospitalization registered in the LHID2005 between 2000 and 2017. The index date was defined as that on which a new diagnosis of hypothyroidism was made. The exclusion criteria were as follows: history of hypothyroidism before the index date, TB before tracking and without tracking, age <20 years, and unknown sex. For purpose 2, we used validated inclusion criteria for TB patients by ICD-9 code of 010-018 and ICD-10 code of A15-A19 plus prescription of at least two anti-TB drugs (e.g., isoniazid, ethambutol, rifampin, pyrazinamide) for 4 weeks within 180 days of TB diagnosis (32) registered in the LHID2005 between 2000 and 2017. TB outside of the lung, such as the lymphatic system, bones and joints, liver, central nervous system, genitourinary, etc., is called extra-pulmonary TB. Miliary TB is classified as extra-pulmonary TB if the patient has no lesions in the lungs. If the patient has both pulmonary and extra-pulmonary TB, the patient is classified as pulmonary TB (33). The exclusion criteria were similar to purpose 1. Four-fold propensity score matching (34) was employed to match the above mentioned study cohorts with the control cohorts selected from the LHID2005 by sex, age, comorbidities, and index date. A flowchart of the selection of study participants is shown in Figure 1.

To further assess the association between TRT and the TB risk in patients with hypothyroidism, subgroup analyses were conducted stratified of data by the duration of TRT (6 weeks−3 months, 3 months−1 year, and ≥1 year). The TRT group was defined as the patients with hypothyroidism who had received levothyroxine sodium prescription for at least 6 weeks during the study period. The non-TRT group included patients with hypothyroidism who had never received levothyroxine sodium prescription or had been on medication for <6 weeks during the study period. The ICD and Anatomical Therapeutic Chemical codes used in this study are presented in Supplementary Table S1.

### Outcomes and Follow-Up

The endpoints of this study were new diagnoses of TB (purpose 1) and hypothyroidism (purpose 2). The definitions of new TB and hypothyroidism cases were the same as the inclusion criteria mentioned above. In Taiwan, clinicians must report new TB cases to the Taiwan Centers for Disease Control within seven days and submit the final histopathology and TB culture results for document review and medical reimbursement. In addition, the high accuracy of TB diagnoses in the LHID has been validated previously (32).

The follow-up period was defined as the time interval from the index date to the diagnosis of TB (purpose 1) or hypothyroidism (purpose 2), 31 December, 2017, or withdrawal from the NHI programme, whichever occurred first.

### Demographic Variables and Comorbidities

Data on demographic variables, including sex, age, insurance premium, and urbanization level, were collected. Comorbidities or possible risk factors for TB or hypothyroidism, including DM (ICD-9: 250, ICD-10: E10-E14), HTN (ICD-9: 401-405, ICD-10: I10-I15), hyperlipidaemia (ICD-9: 272, ICD-10: E74-75, E77-E78, E88), ischaemic heart disease (IHD; ICD-9: 401-414, ICD-10: I20-I25), congestive heart failure (ICD-9: 428, ICD-10: I50), cancer (ICD-9: 140-208, ICD-10: C00-C96), COPD (ICD-9: 490-496, ICD-10: J40-J47), stroke (ICD-9: 430-438, ICD-10: I60-I69), chronic kidney disease (CKD; ICD-9: 585, ICD-10: N18), human immunodeficiency virus (HIV) infection (ICD-9: 042, V08, ICD-10: B20-B24, Z21), and cirrhosis (ICD-9: 517.2, 571.5-571.6, ICD-10: K73-K74, K76), were identified.

### Statistical Analysis

The chi-square test was used to compare the differences in categorical variables between the two purpose and control groups. An independent-samples *t*-test was used for continuous variables. The cumulative risks for TB (purpose 1) and hypothyroidism (purpose 2) were computed using the Kaplan–Meier method, and the log-rank test was used to compare differences between the curves. The incidence rate was calculated per 1000 person-years. The hazard ratios and 95% CI were calculated using the multivariable Cox proportional hazards regression analysis, which was adjusted for sex, age, insured premium, comorbidities, and urbanization level. We further evaluated the interaction effect of hypothyroidism and comorbidities on the risk of TB. All statistical analyses were performed using IBM SPSS Statistics for Windows, version 22.0 (IBM Corp., Armonk, NY, USA), and a two-tailed *P*-value < 0.05 was considered statistically significant.

## Results

### Baseline Characteristics of the Patients in the Study

After 1:4 propensity score matching for sex, age, comorbidities, and index date, we identified 3,976 patients in the hypothyroidism cohort, 15,904 patients in the non-hypothyroidism cohort, 35,120 patients in the TB cohort, and 140,480 patients in the non-TB cohort (Figure 1). Between two purposes and control cohorts, baseline characteristics showed no significant differences in sex, age, and comorbidities (Table 1).

**Table 1:** Baseline characteristics of study participants.

**Variables**	**Risk for TB (purpose 1)**					**Risk for hypothyroidism (purpose 2)**				
	**Hypothyroidism**		**Non-hypothyroidism**		***P***	**TB**		**Non-TB**		***P***
	***n***	**%**	***n***	**%**		***n***	**%**	***n***	**%**	
Total	3,976	20.0	15,904	80.0		35,120	20.0	140,480	80.0	
Sex					0.999					0.999
Male	1,425	35.8	5,700	35.8		19,986	56.9	79,944	56.9	
Female	2,551	64.2	10,204	64.2		15,134	43.1	60,536	43.1	
Mean age	35.4 ± 18.9 (years)		36.0 ± 19.8 (years)		0.074	61.0 ± 19.6 (years)		61.2 ± 20.0 (years)		0.080
Age group (years)					0.999					0.999
20–44	1,865	46.9	7,460	46.9		6,224	17.7	24,896	17.7	
45–64	971	24.4	3,884	24.4		11,249	32.0	44,996	32.0	
65–74	662	16.7	2,648	16.7		6,538	18.6	26,152	18.6	
75–84	303	7.6	1,212	7.6		5,829	16.6	23,316	16.6	
≥85	175	4.4	700	4.4		5,280	15.0	21,120	15.0	
Insured premium (NT$)					0.266					<0.001
<18,000	2,874	72.3	11,562	72.7		26,253	74.8	101,897	72.5	
18,000–34,999	567	14.3	2,346	14.8		4,350	12.4	19,954	14.2	
≥35,000	535	13.5	1,996	12.6		4,517	12.9	18,629	13.3	
Comorbidities										
DM	1,015	25.5	4,022	25.3	0.757	9,786	27.9	39,175	27.9	0.961
HTN	1,345	33.8	5,289	33.3	0.494	12,250	34.9	49,020	34.9	0.960
Hyperlipidemia	134	3.4	542	3.4	0.961	1,546	4.4	6,203	4.4	0.919
IHD	389	9.8	1,584	10.0	0.740	3,389	9.7	13,567	9.7	0.965
CHF	113	2.8	422	2.7	0.511	973	2.8	3,895	2.8	0.983
Cancer	482	12.1	1,902	12.0	0.777	4,267	12.2	17,088	12.2	0.942
COPD	894	22.5	3,587	22.6	0.926	9,022	25.7	36,182	25.8	0.798
Stroke	423	10.6	1622	10.2	0.414	3,537	10.1	14,264	10.2	0.641
CKD	485	12.2	1935	12.2	0.957	4,870	13.9	19,580	13.9	0.730
HIV	76	1.9	298	1.9	0.896	890	2.5	3,442	2.5	0.366
Cirrhosis	252	6.3	1,026	6.5	0.795	2,789	7.9	11,234	8.0	0.731
Urbanization level					0.054					0.009
1 (Highest)	999	25.1	3,920	24.7		9,803	27.9	39,184	27.9	
2	1,135	28.6	4,892	30.8		10,131	28.9	41,225	29.4	
3	864	21.7	3,352	21.1		7,023	20.0	27,030	19.2	
4 (Lowest)	978	24.6	3,740	23.5		8,163	23.2	33,041	23.5	

*P-values were determined using Chi-square/Fisher exact test for categorical variables and independent-samples t-test for continuous variables*.

*CHF, congestive heart failure; CKD, chronic kidney disease; COPD, chronic obstructive pulmonary disease; DM, diabetes mellitus; HIV, human immunodeficiency virus; HTN, hypertension; IHD, ischemic heart disease; TB, tuberculosis*.

### Purpose 1: Hypothyroidism and the Risk of Developing TB

Female was predominated (64.2%) in the purpose 1 and more than half of the participants were age <65 years. At the time of diagnosis of TB, the hypothyroidism cohort had a higher prevalence of DM, HTN, hyperlipidemia, IHD, cancer, COPD, stroke, and CKD than the non-hypothyroidism cohort (Supplementary Table S2). The mean follow-up periods in the hypothyroidism and non-hypothyroidism cohorts were 10.64 and 10.85 years, respectively (Supplementary Table S3). The mean duration before TB development were 7.74 and 8.25 years in the hypothyroidism and non-hypothyroidism cohorts, respectively (Supplementary Table S4).

**Figure 2:**
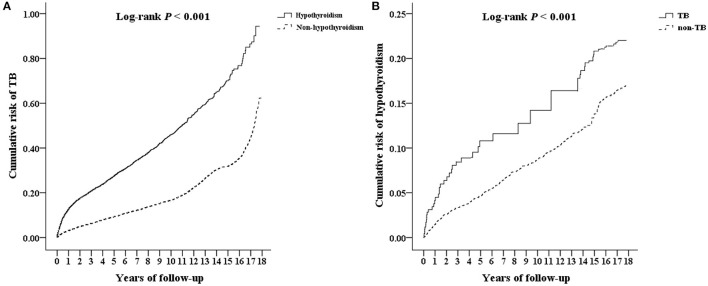
Kaplan–Meier analysis for cumulative risk of developing **(A)** TB and **(B)** hypothyroidism among patients aged ≥20 years using log-rank test. TB, tuberculosis.

Multivariate Cox proportional hazard analysis revealed that the hypothyroidism cohort has a significantly higher risk of developing TB than the non-hypothyroidism cohort (Table 2). Furthermore, the hypothyroidism cohort exhibited a significantly higher risk for TB, considering nearly all variables, i.e., male sex, all age groups, comorbidities (except for congestive heart failure and cancer), and urbanization level (except for level 3). Table 3 demonstrated an interaction effect between hypothyroidism and comorbidities including DM (aHR = 3.46, 95% CI 2.25–4.01) and COPD (aHR = 4.33, 95% CI 2.35–6.11) on the risk of developing TB. At the end of the follow-up period, the hypothyroidism cohort had a higher cumulative risk for TB than those in the non-hypothyroidism cohort (Figure 2A).

**Table 2:** Factors related to events stratified by variables using Cox regression analysis.

**Variables**	**Risk for TB (Purpose 1)**		**Risk for hypothyroidism (Purpose 2)**	
	**aHR^†^ (95% CI)**	***P***	**aHR^†^ (95% CI)**	***P***
Cohort				
Hypothyroidism	2.91 (1.50–3.65)	<0.001		
TB			2.01 (1.41–2.38)	<0.001
Sex				
Male vs. female	1.86 (1.12–2.68)	<0.001	0.84 (0.33–2.00)	0.652
Age group (years)				
20–44	Reference		Reference	
45–64	2.02 (1.55–2.49)	<0.001	1.28 (0.81–1.82)	0.382
65–74	2.77 (2.27–3.26)	<0.001	1.04 (0.57–1.66)	0.424
75–84	2.48 (2.04–3.01)	<0.001	1.01 (0.54–1.59)	0.432
≥85	1.69 (1.21–2.17)	<0.001	1.00 (0.40–1.55)	0.506
Insured premium (NT$)				
<18,000	Reference		Reference	
18,000–34,999	0.97 (0.53–1.34)	0.498	0.88 (0.30–1.54)	0.584
≥35,000	0.85 (0.41–1.18)	0.583	0.70 (0.18–1.37)	0.722
Comorbidities				
DM	2.86 (2.36–3.99)	<0.001	2.64 (1.62–3.74)	<0.001
HTN	2.91 (2.38–4.30)	<0.001	2.40 (1.55–3.24)	<0.001
Hyperlipidemia	1.99 (1.40–2.58)	<0.001	2.27 (1.53–3.05)	<0.001
IHD	1.46 (1.13–1.80)	<0.001	1.98 (1.40–2.58)	<0.001
CHF	1.11 (0.51–1.94)	0.424	1.00 (0.30–2.00)	0.572
Cancer	1.40 (0.97–1.81)	0.095	1.16 (0.66–1.88)	0.382
COPD	2.01 (1.46–2.48)	<0.001	1.39 (1.08–1.88)	<0.001
Stroke	1.92 (1.32–2.49)	<0.001	1.77 (1.24–2.22)	<0.001
CKD	1.72 (1.22–2.46)	<0.001	2.12 (1.42–2.87)	<0.001
HIV	3.02 (2.20–3.59)	<0.001	1.46 (1.12–1.79)	<0.001
Cirrhosis	1.80 (1.21–2.34)	<0.001	1.44 (1.15–1.66)	<0.001
Urbanization level				
1 (Highest)	2.14 (1.28–3.00)	<0.001	1.47 (0.70–2.12)	0.452
2	1.84 (1.19–2.91)	<0.001	1.22 (0.56–1.77)	0.505
3	1.43 (0.61–1.99)	0.472	1.12 (0.51–1.74)	0.522
4 (Lowest)	Reference		Reference	

*CI, confidence interval; CHF, congestive heart failure; CKD, chronic kidney disease; COPD, chronic obstructive pulmonary disease; DM, diabetes mellitus; HIV, human immunodeficiency virus; HTN, hypertension; IHD, ischemic heart disease; TB, tuberculosis*.

† *aHR, adjusted hazard ratio: adjusted for the variables listed in this table by using Cox regression analysis*.

**Table 3:** Cox proportional hazard regression analysis for the hypothyroidism-linked TB risk with the interaction of comorbidity.

**Comorbidities**	**Risk for TB (purpose 1): Hypothyroidism (yes vs. no)**			
	**aHR^†^**	**95% CI**	***P***	***P*** **for interaction**
Overall	2.91	1.50–3.65	<0.001	
DM				0.042
Without	2.04	1.38–2.08	<0.001	
With	3.46	2.25–4.01	<0.001	
HTN				0.556
Without	2.37	1.33–3.53	<0.001	
With	3.12	1.65–3.98	<0.001	
Hyperlipidemia				0.663
Without	2.85	1.47–3.59	<0.001	
With	2.94	1.53–3.69	<0.001	
IHD				0.442
Without	2.16	1.38–3.23	<0.001	
With	3.20	1.64–4.01	<0.001	
CHF				0.971
Without	2.90	1.48–3.62	<0.001	
With	2.92	1.53–3.67	<0.001	
Cancer				0.121
Without	2.72	1.24–3.28	<0.001	
With	3.56	2.06–4.19	<0.001	
COPD				<0.001
Without	1.65	1.18–2.20	<0.001	
With	4.33	2.35–6.11	<0.001	
Stroke				0.517
Without	2.41	1.41–3.56	<0.001	
With	3.11	1.64–3.89	<0.001	
CKD				0.422
Without	2.37	1.41–3.28	<0.001	
With	3.36	1.61–3.94	<0.001	
HIV				0.084
Without	2.03	1.40–2.62	<0.001	
With	4.05	2.42–5.17	<0.001	
Cirrhosis				0.673
Without	2.19	1.34–3.48	<0.001	
With	3.35	1.70–3.80	<0.001	

*CI, confidence interval; CHF, congestive heart failure; CKD, chronic kidney disease; COPD, chronic obstructive pulmonary disease; DM, diabetes mellitus; HIV, human immunodeficiency virus; HTN, hypertension; IHD, ischemic heart disease; TB, tuberculosis*.

*aHR, adjusted hazard ratio: adjusted for the variables listed in Table 2 by using Cox regression analysis*.

The hypothyroidism cohort was stratified according to the duration of TRT (Table 4). Patients with hypothyroidism who had never received TRT during the study period had a 3.62 times higher risk of developing TB than those without hypothyroidism. In the TRT group, the highest risk occurred in the treatment period lasting from 6 weeks to 3 months. The hypothyroidism-linked TB risk may be attenuated gradually by long-term TRT. There was no significant difference between the groups in the treatment categories with ≥1-year treatment period. The hypothyroidism cohort had a significantly higher aHR for TB during the follow-up periods of <3 months, 3–6 months, 6–12 months, and 1–5 years than the non-hypothyroidism cohort (Table 6).

**Table 4:** Adjusted hazard ratios of developing TB in hypothyroidism cohort (purpose 1).

**Cohort**	**Population**	**Overall TB event**			**Pulmonary TB event**			**Extra-pulmonary TB event**		
		***n***	**Rate^†^**	**aHR^‡^ (95% CI)**	***n***	**Rate^†^**	**aHR^‡^ (95% CI)**	***n***	**Rate^†^**	**aHR^‡^ (95% CI)**
Non-hypothyroidism	15,904	717	4.16	Reference	647	3.75	Reference	70	0.41	Reference
Hypothyroidism^**§**^	3,976	233	5.51	2.91*** (1.50–3.65)	209	4.94	2.90*** (1.50–3.62)	24	0.57	3.07*** (1.59–3.85)
Non-TRT (including <6 weeks)	690	54	7.52	3.62*** (2.19–4.84)	46	6.41	3.76*** (1.94–4.70)	8	1.11	6.04*** (3.12–6.96)
TRT	3,286	179	5.10	2.40*** (1.26–3.01)	163	4.64	2.72*** (1.40–3.40)	16	0.46	2.47*** (1.27–3.09)
6 weeks−3 months	1,073	74	6.61	2.98*** (1.54–3.73)	68	6.08	3.56*** (1.84–4.46)	6	0.54	2.90*** (1.50–3.64)
3 months−1 year	1,124	60	4.76	2.33*** (1.23–2.95)	54	4.28	2.51*** (1.30–3.14)	6	0.48	2.58*** (1.33–3.23)
≥1 year	1,089	45	3.97	1.91 (0.95–2.39)	41	3.62	2.02 (0.97–2.68)	4	0.35	1.91 (0.99–2.39)

*CI, confidence interval; TB, tuberculosis; TRT, thyroxine replacement treatment*.

*** *P < 0.001*.

† *Incidence rate, per 1,000 person-years*.

‡ *aHR, adjusted hazard ratio: adjusted for the variables listed in Table 2 by using Cox regression analysis*.

§ *Hypothyroidism cohort is stratified by duration of TRT (non-TRT, 6 weeks to 3 months, 3 months to 1 year, and ≥1 year)*.

### Purpose 2: TB and the Risk of Developing Hypothyroidism

Male was predominated (56.9%) in the purpose 2 and more than half of the participants were age ≥ 65 years. At the time of diagnosis of TB, the TB cohort had a higher prevalence of DM, HTN, hyperlipidemia, and IHD than the non-TB cohort (Supplementary Table S2). The mean follow-up periods in the TB and non-TB cohorts were 10.89 and 10.97 years, respectively (Supplementary Table S3). The mean period before hypothyroidism development was 7.30 years in the TB cohort and 8.18 years in the non-TB cohort (Supplementary Table S4).

According to the multivariate Cox proportional hazard analysis, the aHR for the risk of developing hypothyroidism was 2.01 times higher in the TB cohort than in the non-TB cohort (Table 2). After adjusting for variables, patients with comorbidities (DM, HTN, hyperlipidaemia, IHD, COPD, stroke, CKD, HIV, and cirrhosis) in the TB cohort had a significantly higher aHR for hypothyroidism than those in the non-TB cohort. The TB cohort had a significantly higher cumulative risk of developing hypothyroidism than the non-TB cohort at the end of the follow-up period (Figure 2B).

The subgroup analysis showed that patients with pulmonary TB and extra-pulmonary TB have a significantly higher aHR for developing hypothyroidism than those without TB (Table 5). Furthermore, the TB cohort had a significantly higher aHR for hypothyroidism in the follow-up periods of <3, 3–6, 6–12 months, and 1–5 years than the non-TB cohort (Table 6).

**Table 5:** Adjusted hazard ratios of developing hypothyroidism in TB cohort (purpose 2).

**Cohort**	**Population**	**Hypothyroidism event**		
		***n***	**Rate^†^**	**aHR^‡^ (95% CI)**
Non-TB	140,480	1,068	0.74	Reference
TB^§^	35,120	379	1.00	2.01^***^ (1.41–2.38)
Pulmonary TB	32,900	359	1.01	2.04^***^ (1.43–2.40)
Extra-pulmonary TB	2,220	20	0.83	1.68^***^ (1.19–1.98)

*CI, confidence interval; TB, tuberculosis*.

*** *P < 0.001*.

† *Incidence rate, per 1,000 person-years*.

‡ *aHR, adjusted hazard ratio: adjusted for the variables listed in Table 2 by using Cox regression analysis*.

§ *TB cohort is stratified by classification of TB*.

**Table 6:** Adjusted hazard ratios of developing TB/hypothyroidism in different follow-up periods.

**Follow-up period**	**Risk for TB (purpose 1): Hypothyroidism (yes vs. no)**			**Risk for hypothyroidism (purpose 2): TB (yes vs. no)**		
	**Population**	**aHR^**†**^ (95% CI)**	***P***	**Population**	**aHR^**†**^ (95% CI)**	***P***
Overall	19,880	2.91 (1.50–3.65)	<0.001	175,600	2.01 (1.41–2.38)	<0.001
<3 months	1,835	2.95 (1.52–3.69)	<0.001	16,305	2.04 (1.43–2.41)	<0.001
3–6 months	1,420	3.60 (1.86–4.50)	<0.001	13,441	2.49 (1.74–2.94)	<0.001
6 months−1 year	2,133	3.27 (1.69–4.10)	<0.001	18,729	2.26 (1.58–2.68)	<0.001
1–5 years	6,011	2.87 (1.49–3.60)	<0.001	54,117	1.99 (1.39–2.35)	<0.001
5–8 years	4,101	2.02 (0.99–2.41)	0.068	36,010	1.33 (0.93–1.57)	0.072
≥8 years	4,380	1.78 (0.92–2.24)	0.111	36,998	1.23 (0.87–1.46)	0.230

*CI, confidence interval; TB, tuberculosis*.

*aHR, adjusted hazard ratio: adjusted for the variables listed in Table 2 using Cox regression analysis*.

## Discussion

To the best of our knowledge, this is the first longitudinal, nationwide, population-based cohort study to investigate the bidirectional relationship between hypothyroidism and TB. We found that patients with hypothyroidism have a 2.91 times higher risk of developing TB than those without hypothyroidism, and that patients with TB have a 2.01 times higher risk of developing hypothyroidism than that those without. Although the pathophysiological association between hypothyroidism and tuberculosis remains unclear, several hypotheses have been proposed.

In our data, hypothyroidism was predominated in the female (64.2%). But male with hypothyroidism had a higher risk of TB development, especially in the age ≥45 years. Besides, the higher urbanization level 1 and 2 also had an impact on TB development. We proposed some explanations for these interesting findings. First, previous studies revealed that male and old age were the risk factors of TB development (35, 36). Testosterone can impair macrophage activation and decrease the production of proinflammatory cytokines, which may increase the susceptibility to Mtb infection. In contrast, estrogen can enhance macrophage activation and induce proinflammatory cytokines, which provide protection against Mtb infection (37). Second, a modeling study in Taiwan showed that immune senescence played an important role in the age disparity between children and elders, which means patients with latent TB infection have an increased risk of developing TB as they age due to a weakening immune system with older age (38). Third, TB is a disease whose transmission is favored by the density of urban populations (39). Taiwan has seen rapid growth in living standards and nationwide coverage of high-quality, publicly funded healthcare services (28). Due to the patient preference, better service quality, increased geographic accessibility, better community awareness, and access to better diagnostics and treatment, the TB case notification rates are higher in urban areas than in rural areas (39). Our findings are compatible with the above explanations.

In Taiwan, more than half of the new TB cases occur in the elderly population (age ≥ 65 years) since 2005 (3) and it can be seen in our TB cohort. In general concept, hypothyroidism development is much more common in female than male, and the incidence rate increase with age (40). However, these findings were not seen in our study. We think there may be other possible causes, with drug-induced hypothyroidism being one of the most likely, and additional study is needed in the future. Besides, this study also showed that patients with hypothyroidism have an interaction effect with comorbidities including DM and COPD toward the risk of developing TB. Individual links between TB and these two comorbidities among the general population are well established among papers (41). This demonstrates a relationship between hypothyroidism and DM and COPD and how all these interrelations remain unexplored.

Previous studies have demonstrated that serum triiodothyronine (T3) and thyroxine (T4) modulate specific immune responses, including innate and adaptive immune responses, which change significantly during aging and in cases of hypothyroidism. The dysregulation of innate and adaptive immune responses may subsequently increase susceptibility to infection (42–44). A recent population-based cohort study showed that patients with hypothyroidism were associated with a risk of pneumonia. In addition, the use of TRT (>30 days) can attenuate the hypothyroidism-linked pneumonia risk (45). However, there was no evidence or mechanism to explain the TB-linked hypothyroidism. In a rabbit Mtb infection model, defective production of thyroid hormones increased susceptibility to Mtb infection (46). Furthermore, T3 and T4 restricted Mtb growth in human monocyte-derived macrophages through interleukin-1α production; therefore, young, healthy household contacts of patients with TB with a decreased production of T4 at baseline have an increased risk of developing active TB (20). Our findings strengthen the evidence that patients with hypothyroidism have an increased risk of developing TB. The non-TRT group showed a significantly higher risk of developing TB, especially pulmonary TB, than the non-hypothyroidism cohort. Additionally, we found that long-term TRT (>1 year) may decrease the risk of developing TB, which supports the findings of a previous study (20). Further prospective cohort studies to evaluate the association between TRT and the risk of TB are warranted.

In our study, patients with TB, pulmonary TB, or extra-pulmonary TB had a significantly higher risk of developing hypothyroidism than those without TB. However, the results of previous, similar studies (20, 47) have been controversial. Kleynhans investigated the changes in the immune system during treatment for TB in patients who were cured and in those in whom it failed. They found that T3 concentrations increase during TB infection, whereas T4 concentrations remain unchanged in the failed group. T4 concentrations were lower in the cured group after 6 months of TB treatment than in the failed group. T4 plays a critical role in Mtb infection and may be a potential biomarker to differentiate treatment outcomes (47). Recently, T3 and T4 concentrations were reported to be elevated in TB progressors (healthy household contacts developing TB during the follow-up period) after 6 months of TB treatment, although T4 concentrations decreased in treated TB progressors 3 years after TB treatment (20). Although previous studies have demonstrated an association between TB and thyroid hormone levels, our findings reinforce that TB is a risk factor for hypothyroidism.

Moreover, we observed that the TB and hypothyroidism cohorts have a higher risk for hypothyroidism and TB, respectively during a follow-up period of <5 years; the highest risks were noted in the 3–6 month follow-up. To our knowledge, this is the first study to reveal this correlation. We proposed possible explanations for the short-term and long-term risks of this observation. First, rifampin enhances T4 clearance and may cause hypothyroidism (16, 17). Without treatment, ~4–15% of patients with latent TB infection develop TB within 1–5 years after getting infected (48–50). Devalraju et al. found that 17 of 688 healthy household contacts developed active TB, and that 12 of 17 progressors received a diagnosis of TB within the first year (20). These may be the reasons for the short-term risk of both diseases. Second, Mtb infection may trigger autoimmune reactions by molecular mimicry of Mtb antigens with human proteins (51) and infection may subsequently result in autoimmune thyroid diseases (22). Third, asymptomatic populations of hypothyroidism and TB may result in delayed diagnosis. And finally, genetic polymorphism plays a major role in the progression to active TB (52) and two single nucleotide polymorphisms in immune-and inflammation-related genes (interleukin-6 rs2066992 and rs1524107) increased the risk of active TB (53). In addition, interleukin-6 can inhibit thyroid function *in vivo* (54, 55). The relationship between the long-term risk of TB and hypothyroidism may involve complex interactions between genes, cytokines, and the immune system, and additional studies are warranted to elucidate these mechanisms.

Our study had some limitations. First, we were unable to analyze the type or disease severity of hypothyroidism and TB in both cohorts because of the lack of symptoms and the absence of associated laboratory data, such as concentrations of thyroid-stimulating hormone, free T4, T3, or anti-thyroid peroxidase antibodies, in the NHIRD. Second, similar to issues with all electronic health databases, there can be problems related to coding errors and deliberate upcoding. Although previous studies have demonstrated high accuracy and validity for ICD-9-CM-based LHID disease identification (27, 29–31), we sought to reduce misclassification bias with a stricter definition of hypothyroidism and TB (29). On the other hand, we may underestimate the populations of asymptomatic subclinical hypothyroidism. Third, clinicians did not routinely examine chest radiography scans and thyroid functions in either cohort, which might have resulted in underdiagnosis. Fourth, although we adjusted for the latent variables in the table, there might have been unknown or unmeasured confounding biases. Fifth, although rifampin, ethionamide and para-aminosalicylic acid were the possible cause of hypothyroidism (16, 17, 23), we did not perform an analysis of these drugs. Additional research should be performed in the future.

In conclusion, the current study revealed a bidirectional relationship between TB and hypothyroidism. Patients with hypothyroidism showed a 2.91-fold higher risk of developing TB than the general population, especially those combined with DM or COPD. The hypothyroidism-linked TB risk may be attenuated by long-term TRT. Physicians should be aware of the risk of developing hypothyroidism when treating patients with TB. We recommend that public health authorities should conduct surveillance for both diseases to reduce the associated disease burden.

## Data Availability Statement

The data analyzed in this study is subject to the following licenses/restrictions: The data on the study population that were obtained from the NHIRD are maintained in the NHIRD. The National Health Research Institutes (NHRI) is a non-profit foundation established by the government. Only citizens of Taiwan who fulfill the requirements of conducting research projects are eligible to apply for access to the NHIRD. The use of the NHIRD is limited to research purposes only. Applicants must follow the Computer-Processed Personal Data Protection Law and the related regulations of the National Health Insurance Administration and NHRI, and an agreement must be signed by the applicant and their supervisor upon application submission. All applications are reviewed for approval of data release. Requests to access these datasets should be directed to https://nhird.nhri.org.tw/.

## Ethics Statement

The studies involving human participants were reviewed and approved by Institutional Review Board of the Tri-Service General Hospital (approval number: B-111-01). Written informed consent for participation was not required for this study in accordance with the national legislation and the institutional requirements.

## Author Contributions

L-TC, C-CS, S-HW, and S-ET contributed to conception and design of the study. C-HC and W-CC organized the database and performed the statistical analysis. L-TC wrote the first draft of the manuscript. C-KP, S-HW, and S-ET wrote sections of the manuscript. L-TC, S-YW, G-JW, C-HT, C-AS, and S-ET were responsible for the critical revision of the manuscript for important intellectual content. All authors read and approved the final manuscript.

## Funding

This study was supported by the Tri-Service General Hospital Research Foundation (TSGH-E-111230), the sponsor had no role in the study design, data collection, analysis, decision to publish, or preparation of the manuscript.

## Conflict of Interest

The authors declare that the research was conducted in the absence of any commercial or financial relationships that could be construed as a potential conflict of interest.

## Publisher's Note

All claims expressed in this article are solely those of the authors and do not necessarily represent those of their affiliated organizations, or those of the publisher, the editors and the reviewers. Any product that may be evaluated in this article, or claim that may be made by its manufacturer, is not guaranteed or endorsed by the publisher.

## Abbreviations

aHR: Adjusted hazard ratio
CI: confidence interval
CKD: chronic kidney disease
COPD: chronic obstructive pulmonary disease
DM: diabetes mellitus
HIV: human immunodeficiency virus
HTN: hypertension
ICD-9-CM, International Classification of Disease, 9th Revision: Clinical Modification
IHD: ischaemic heart disease
LHID2005: Longitudinal Health Insurance Database 2005
Mtb: Mycobacterium tuberculosis
NHI: National Health Insurance
NHIRD: National Health Insurance Research Database
TB: tuberculosis
T3: triiodothyronine
T4: thyroxine.
