# Supplementary material for: Bidirectional Relationship Between Tuberculosis and Hypothyroidism: An 18-Year Nationwide Population-Based Longitudinal Cohort Study
Source: Front Med (Lausanne). 2022 Jul 12;9:900858. doi: 10.3389/fmed.2022.900858 (PMC9320323; doi:10.3389/fmed.2022.900858)
Supplement: Supplementary file 1 [file Data_Sheet_1.docx]

Supplementary Material

# Supplementary Tables

| **Table S1. List of ICD-9-CM, ICD-10-CM, and ATC-code** | | |
| --- | --- | --- |
|  | **Abbreviation** | **ICD-9-CM / ICD-10-CM / ATC-code** |
| **Cohorts, events:** |  |  |
| Hypothyroidism |  | 243-244 / E00, E01.8, E02-E03, E89.0; ≥3 outpatient visits or inpatient |
| Levothyroxine sodium |  | H03AA01; ≥6 weeks |
| Tuberculosis | TB | 010-018 / A15-A19; ≥2 anti-TB drugs for 4 weeks within 180 days of TB diagnosis |
| Pulmonary TB |  | 010-011/ A15.0-A15.3, A15.7, A16.0-A16.3, A16.7 |
| Extra-pulmonary TB |  | 012-018/ A15.4-A15.6, A15.8, A15.9, A16.4-A16.6, A16.8, A16.9, A17-A19 |
| **Comorbidities:** |  |  |
| Diabetes mellitus | DM | 250 / E10-E14 |
| Hypertension | HTN | 401-405 / I10-I15 |
| Hyperlipidemia |  | 272 / E74-E75, E77-E78, E88 |
| Ischemic heart disease | IHD | 410-414 / I20-I25 |
| Congestive heart failure | CHF | 428 / I50 |
| Cancer |  | 140-208 / C00-C96 |
| Chronic obstructive pulmonary disease | COPD | 490-496 / J40-J47 |
| Stroke |  | 430-438 / I60-I69 |
| Chronic kidney disease | CKD | 585 / N18 |
| Human immunodeficiency virus | HIV | 042, V08 / B20-B24, Z21 |
| Cirrhosis |  | 571.2, 571.5-571.6 / K73-K74, K76 |

**Table S2.** Characteristics of study participants at the time of diagnosis of TB (purpose 1) and hypothyroidism (purpose 2)

| **Variables** | **Risk for TB (purpose 1)** | | | | | |  | **Risk for hypothyroidism (purpose 2)** | | | | | | |
| --- | --- | --- | --- | --- | --- | --- | --- | --- | --- | --- | --- | --- | --- | --- |
|  | **Hypothyroidism** | |  | **Non-hypothyroidism** | |  |  | **TB** | |  | **Non-TB** | |  |  |
|  | ***n*** | **%** |  | ***n*** | **%** | ***P*** |  | ***n*** | **%** |  | ***n*** | **%** | ***P*** |  |
| Total | 3976 | 20.0 |  | 15 904 | 80.0 |  |  | 35 120 | 20.0 |  | 140 480 | 80.0 |  |  |
| Events |  |  |  |  |  | <0.001 |  |  |  |  |  |  | <0.001 |  |
| With | 233 | 5.9 |  | 717 | 4.5 |  |  | 379 | 1.1 |  | 1068 | 0.8 |  |  |
| Without | 3743 | 94.1 |  | 15 187 | 95.5 |  |  | 34 741 | 98.9 |  | 139 412 | 99.2 |  |  |
| Sex |  |  |  |  |  | 0.999 |  |  |  |  |  |  | 0.999 |  |
| Male | 1425 | 35.8 |  | 5700 | 35.8 |  |  | 19 986 | 56.9 |  | 79 944 | 56.9 |  |  |
| Female | 2551 | 64.2 |  | 10 204 | 64.2 |  |  | 15 134 | 43.1 |  | 60 536 | 43.1 |  |  |
| Mean age | 46.1±19.2 (years) | |  | 46.9±20.0 (years) | | 0.019 |  | 71.9±19.7 (years) | |  | 72.2±20.3 (years) | | 0.016 |  |
| Age group (years) |  |  |  |  |  | 0.026 |  |  |  |  |  |  | 0.005 |  |
| 20‒44 | 1752 | 44.1 |  | 7003 | 44.0 |  |  | 6123 | 17.4 |  | 24 412 | 17.4 |  |  |
| 45‒64 | 964 | 24.3 |  | 3825 | 24.1 |  |  | 11 224 | 32.0 |  | 43 905 | 31.3 |  |  |
| 65‒74 | 633 | 15.9 |  | 2511 | 15.8 |  |  | 6518 | 18.6 |  | 26 027 | 18.5 |  |  |
| 75‒84 | 304 | 7.7 |  | 1216 | 7.7 |  |  | 5842 | 16.6 |  | 23 402 | 16.7 |  |  |
| ≥85 | 323 | 8.1 |  | 1349 | 8.5 |  |  | 5413 | 15.4 |  | 22 734 | 16.2 |  |  |
| Insured premium (NT$) |  |  |  |  |  | 0.266 |  |  |  |  |  |  | <0.001 |  |
| <18 000 | 2874 | 72.3 |  | 11 562 | 72.7 |  |  | 26 253 | 74.8 |  | 101 897 | 72.5 |  |  |
| 18 000‒34 999 | 567 | 14.3 |  | 2346 | 14.8 |  |  | 4350 | 12.4 |  | 19 954 | 14.2 |  |  |
| ≥35 000 | 535 | 13.5 |  | 1996 | 12.6 |  |  | 4517 | 12.9 |  | 18 629 | 13.3 |  |  |
| Comorbidities |  |  |  |  |  |  |  |  |  |  |  |  |  |  |
| DM | 1245 | 31.3 |  | 4068 | 25.6 | <0.001 |  | 10 124 | 28.8 |  | 39 375 | 28.0 | 0.003 |  |
| HTN | 1597 | 40.2 |  | 5304 | 33.4 | <0.001 |  | 13 454 | 38.3 |  | 50 003 | 35.6 | <0.001 |  |
| Hyperlipidemia | 172 | 4.3 |  | 551 | 3.5 | 0.011 |  | 1678 | 4.8 |  | 6211 | 4.4 | 0.004 |  |
| IHD | 482 | 12.1 |  | 1593 | 10.0 | <0.001 |  | 3526 | 10.0 |  | 13 606 | 9.7 | 0.045 |  |
| CHF | 124 | 3.1 |  | 430 | 2.7 | 0.161 |  | 988 | 2.8 |  | 3904 | 2.8 | 0.731 |  |
| Cancer | 572 | 14.4 |  | 2021 | 12.7 | 0.005 |  | 4305 | 12.3 |  | 17 104 | 12.2 | 0.642 |  |
| COPD | 1034 | 26.0 |  | 3592 | 22.6 | <0.001 |  | 9077 | 25.9 |  | 36 198 | 25.8 | 0.764 |  |
| Stroke | 488 | 12.3 |  | 1644 | 10.3 | <0.001 |  | 3631 | 10.3 |  | 14 288 | 10.2 | 0.352 |  |
| CKD | 579 | 14.6 |  | 2011 | 12.6 | 0.001 |  | 4892 | 13.9 |  | 19 601 | 14.0 | 0.910 |  |
| HIV | 88 | 2.2 |  | 300 | 1.9 | 0.179 |  | 907 | 2.6 |  | 3444 | 2.5 | 0.161 |  |
| Cirrhosis | 286 | 7.2 |  | 1034 | 6.5 | 0.117 |  | 2864 | 8.2 |  | 11 450 | 8.2 | 0.979 |  |
| Urbanization level |  |  |  |  |  | 0.211 |  |  |  |  |  |  | <0.001 |  |
| 1 (Highest) | 1014 | 25.5 |  | 3982 | 25.0 |  |  | 9902 | 28.2 |  | 39 204 | 27.9 |  |  |
| 2 | 1125 | 28.3 |  | 4725 | 29.7 |  |  | 10 045 | 28.6 |  | 41 102 | 29.3 |  |  |
| 3 | 897 | 22.6 |  | 3402 | 21.4 |  |  | 7086 | 20.2 |  | 27 088 | 19.3 |  |  |
| 4 (Lowest) | 940 | 23.6 |  | 3795 | 23.9 |  |  | 8087 | 23.0 |  | 33 086 | 23.6 |  |  |

*P* values were determined using Chi-square/Fisher exact test for categorical variables and independent-samples *t*-test for continuous variables.

CHF, congestive heart failure; CKD, chronic kidney disease; COPD, chronic obstructive pulmonary disease; DM, diabetes mellitus; HIV, human immunodeficiency virus; HTN, hypertension; IHD, ischemic heart disease; TB, tuberculosis.

**Table S3. Years of follow-up**

| **Purpose** | **Cohort** | **Min** | **Median** | **Max** | **Mean ± SD** | ***P*** |
| --- | --- | --- | --- | --- | --- | --- |
| Risk for TB | Hypothyroidism | 0.01 | 8.87 | 17.96 | 10.64 ± 9.44 |  |
|  | Non-hypothyroidism | 0.01 | 9.06 | 17.97 | 10.85 ± 9.68 |  |
|  | Total | 0.01 | 8.99 | 17.97 | 10.81 ± 9.63 | 0.219 |
| Risk for hypothyroidism | TB | 0.01 | 9.02 | 17.98 | 10.89 ± 9.72 |  |
|  | Non-TB | 0.01 | 9.15 | 17.97 | 10.97 ± 9.95 |  |
|  | Total | 0.01 | 9.11 | 17.97 | 10.96 ± 9.90 | 0.176 |

*P*, independent-samples *t-*test; SD, standard deviation.

**Table S4. Years to events**

| **Purpose** | **Cohort** | **Min** | **Median** | **Max** | **Mean ± SD** | ***P*** |
| --- | --- | --- | --- | --- | --- | --- |
| Risk for TB | Hypothyroidism | 0.02 | 7.25 | 17.36 | 7.74 ± 6.72 |  |
|  | Non-hypothyroidism | 0.02 | 7.94 | 17.82 | 8.25 ± 7.14 |  |
|  | Total | 0.02 | 7.83 | 17.82 | 8.15 ± 7.06 | <0.001 |
| Risk for hypothyroidism | TB | 0.02 | 7.01 | 17.45 | 7.30 ± 6.36 |  |
|  | Non-TB | 0.02 | 7.79 | 17.89 | 8.18 ± 7.09 |  |
|  | Total | 0.02 | 7.62 | 17.89 | 8.00 ± 6.96 | <0.001 |

*P*, independent-samples *t-*test; SD, standard deviation.
